# Influence of Deep Margin Elevation Technique With Two Restorative Materials on Stress Distribution of e.max Endocrown Restorations: A Finite Element Analysis

**DOI:** 10.1155/ijod/6753069

**Published:** 2024-11-27

**Authors:** Fariba MahmoudiYamchi, Mahdi Abbasi, Faezeh Atri, Elham Ahmadi

**Affiliations:** ^1^Dental Research Center, Dentistry Research Institute, School of Dentistry, Tehran University of Medical Sciences, Tehran, Iran; ^2^Dental Research Center, Dentistry Research Institute, Department of Operative Dentistry, School of Dentistry, Tehran University of Medical Sciences, Tehran, Iran; ^3^Department of Prosthodontics, School of Dentistry, Craniomaxillofacial Research Center, Tehran University of Medical Sciences, Tehran, Iran; ^4^Dental Research Center, Dentistry Research Institute, Department of Operative Dentistry, School of Dentistry, Tehran University of Medical Sciences, Tehran, Iran

**Keywords:** deep margin elevation, dental marginal adaptation, dental restoration, dental stress analysis, endocrown, finite element analysis

## Abstract

**Objective:** The impact of the deep margin elevation (DME) technique and its associated materials on the stress distribution in ceramic endocrowns remains to be fully understood. This finite element analysis (FEA) aimed to assess the effects of flowable composite and resin-modified glass ionomer (RMGI) as DME materials on the maximum Von Mises stress (VMS) values and overall stress distribution within ceramic endocrowns and the surrounding tooth structure.

**Materials and Methods:** A mandibular molar featuring a class II mesio occlusal (MO) cavity with the gingival margin of the mesial cavity positioned 2 mm below the cementoenamel junction (CEJ) was prepared and scanned using a Medit i500 scanner. The digital file was then transferred to computer-aided design (CAD) software to create the models. The study generated four scenarios: an intact tooth model (model of intact tooth (MIT)), a prepared tooth model without a DME layer (model without DME (MWD)), a model with a 2 mm DME layer using composite material (model with DME of composite (MDC)), and a model employing RMGI (model with DME of RMGI (MDR)). Stress distribution under axial loads was evaluated based on the Von Mises criterion.

**Results:** The MIT model demonstrated the highest stress concentration at the CEJ region yet exhibited lower stress levels than others. The MWD model showed the highest stress levels. No significant differences in stress distribution patterns were observed between the MDR and MDC models. All models displayed similar stress distributions in the bone.

**Conclusion:** Regardless of the material used, incorporating a DME layer in cavities extending below the CEJ is advisable to achieve uniform stress distribution. Minimizing tooth preparation and preserving tooth structure are recommended.

**Clinical Significance:** Employing a DME layer in cavities with margins below the CEJ is beneficial for reducing stress, irrespective of the material choice.

## 1. Introduction

Restoring large cavities on posterior teeth, particularly following root canal therapy, presents a significant challenge in dentistry. When the margin of these cavities extends below the cementoenamel junction (CEJ), two main issues arise: technical and biological. Technically, subgingival preparation, impression-making, and cementation become problematic. Biologically, such cavities can lead to a reduction in biological width. Historically, two methods—surgical crown lengthening and orthodontic extrusion were employed to address these problems, each with significant drawbacks, including biological complications from crown lengthening and the esthetic and time-consuming nature of orthodontic extrusion [1, 2].

In response to these challenges, clinicians have increasingly turned to the deep margin elevation (DME) technique, pioneered by Dietschi and Spreafico [3]. This method involves elevating the subgingival margins using restorative materials such as flowable composite, conventional packable composite, glass ionomer, and resin-modified glass ionomer (RMGI), thereby, creating a new margin for indirect restoration [4]. This noninvasive and straightforward approach simplifies isolation and impression-making and effectively addresses several clinical issues [5].

Among the various materials available for DME, flowable composite is preferred for its low elastic modulus, minimal polymerization shrinkage, and ease of application in subgingival areas [4]. Conversely, RMGI is favored for its excellent mechanical properties, ability to chemically bond with dentin, fluoride release, high biocompatibility, and thermal expansion coefficient closely matching tooth structure [6–8]. These attributes have guided the choice of these two materials for comparison in this study.

Advancements in dental science and technology continually expand the options available for tooth restoration. Ceramic endocrowns represent a growing trend among these options due to their simplicity, esthetic benefits, strong adhesion, and conservative approach toward tooth preparation. Extensive research supports the use of endocrowns, particularly in endodontically treated teeth.

Lithium disilicate ceramics are commonly used for fabricating endocrowns owing to their excellent esthetic properties, combined micromechanical and macromechanical bonding capabilities, and superior strength and durability [9, 10].

Despite extensive studies into various DME materials and restorative techniques, research comparing the stress distribution across different types of DME materials within the context of endocrown applications has been limited. Moreover, the interplay between DME techniques and endocrown restorations has not been thoroughly investigated, which is especially pertinent given the current popularity of endocrowns.

The finite element method (FEM) has been widely adopted for solving biomechanical issues and analyzing stress distribution influenced by surface geometry, boundary conditions, material properties, and loading scenarios [9]. Consequently, this finite element analysis (FEA) aims to evaluate flowable composite and RMGI as DME materials and to explore the effects of DME on the stress distribution patterns in endocrown restorations and tooth structures. This study proposes two null hypotheses: first, that the application of the DME technique does not affect the stress distribution in the tooth structure and restoration and second, there is no difference in performance between the two DME materials.

## 2. Materials and Methods

**Figure 1:**
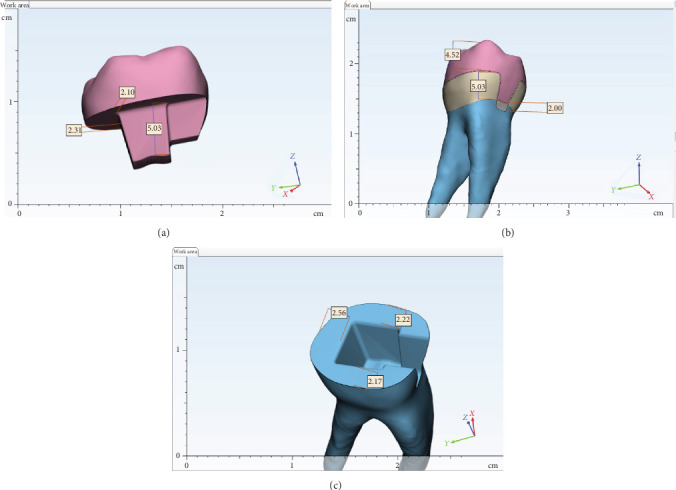
Detailed dimention of prepared endocrown and tooth structure: (A) inner dimensions of endocrown, (B) outer dimension of endocrown and tooth, and (C) thickness of remaining dentin.

A single intact human lower first molar, extracted due to periodontal concerns, was preserved in distilled water following disinfection using an ultrasonic device. The selected tooth was examined and confirmed healthy, devoid of demineralization, cracks, or carious lesions. Initially, the tooth underwent scanning with an intraoral scanner (Medit (i500)) to generate a standard tessellation language (STL) file as a reference model. To simulate a class II cavity preparation, the mesial wall was reduced by 2 mm below the CEJ, achieving a 4 mm buccolingual dimension. Preparation for an endocrown restoration involved reducing the occlusal surface to near half of tooth height using a round-end taper diamond rotary instrument (806314290544; Jota AG), ensuring a flat and stable base for the endocrown. Subsequently, axial preparation was performed to eliminate undercuts without altering the natural geometry of the pulp chamber floor and preparing the internal walls to achieve a 6° divergence [11] with a 5 mm depth into the pulp chamber. Thickness of remaining dentin around endocrown preparation were about 2–2.5 mm (Figure 1). The prepared surfaces were then polished to completion using a fine rotary instrument (806314290504; Jota AG). Then, the endodontic treatment of the molar tooth was done.

The prepared tooth underwent a second scanning process identical to the unaltered tooth, and both STL files were imported into design software (Mimics; Materialize 3-mathics). A three-dimensional (3D) model was created using the scanned references and anatomical landmarks. The reference model for the sound tooth scan file incorporated layers representing enamel, dentin, the pulp chamber, cementum, and the periodontal ligament (PDL) with a thickness of 0.2 mm, as well as spongy and cortical bones with a thickness of 2 mm, along with the corresponding lamina dura.

To prepare the second model (model without DME (MWD)), necessary adjustments to the second scan file have included smoothing all sharp line angles and removing the pulp from the canals. The canals were filled with gutta-percha. An endocrown restoration (Lithium disilicate glass ceramic, innovative pressed system (IPS) e.max computer-aided design (CAD); Ivoclar Vivadent) was then designed, incorporating a cement layer of 0.12 mm (Panavia F2; Kuraray Medical Inc). In this model, the mesial portion of the endocrown extended 2 mm below the CEJ to accommodate cavity extension.

**Figure 2:**
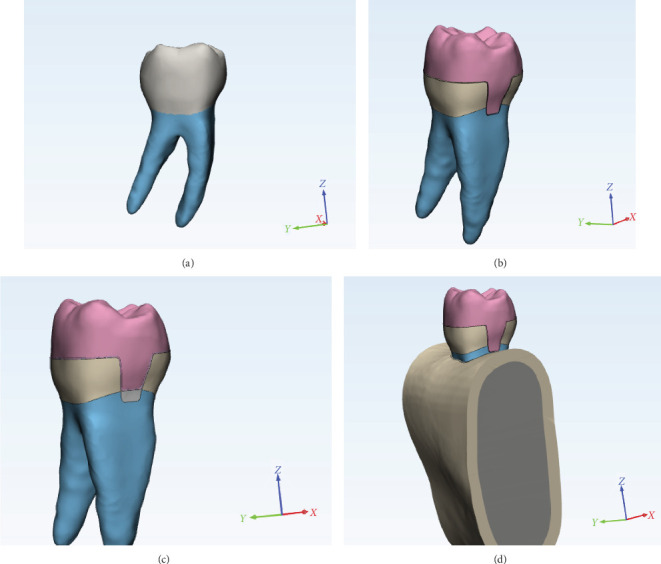
(A) Model of intact tooth. (B) Prepared tooth and endocrown restoration without DME layer. (C) Prepared tooth with DME layer and endocrown restoration. (D) Cortical and spongy bone surround tooth. DME, deep margin elevation.

In the preparation of the third modified model with DME of composite (MDC) and fourth modified model with DME of RMGI (MDR) models, the height of the mesial wall in the MWD model was increased by 2 mm with a layer of DME. Subsequently, an endocrown restoration was designed, featuring a cement layer of 0.12 mm. The mesial portion of the endocrown in these models terminated at the CEJ, supported by a DME layer. The materials used for this DME layer differed between models; a flowable composite for the MDC model and an RMGI for the MDR model. Since this study focused on the evaluation of these materials' mechanical performance in the context of DME and as recent literature showed that the adhesive layer under the composite does not significantly affect the stress distribution on both the tooth and composite resin [12], we ignored this layer in MDC. The periodontium details for these models were consistent with those of the initial reference model (model of intact tooth (MIT); Figure 2).

All materials utilized in the models were considered homogeneous, elastic, and isotropic.  Table 1 enumerates the Poisson's ratio and modulus of elasticity for all materials used.

**Figure 3:**
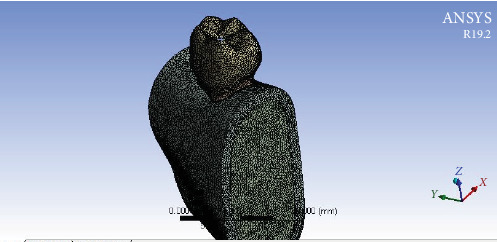
Mesh refinement with tetrahedral elements.

**Figure 4:**
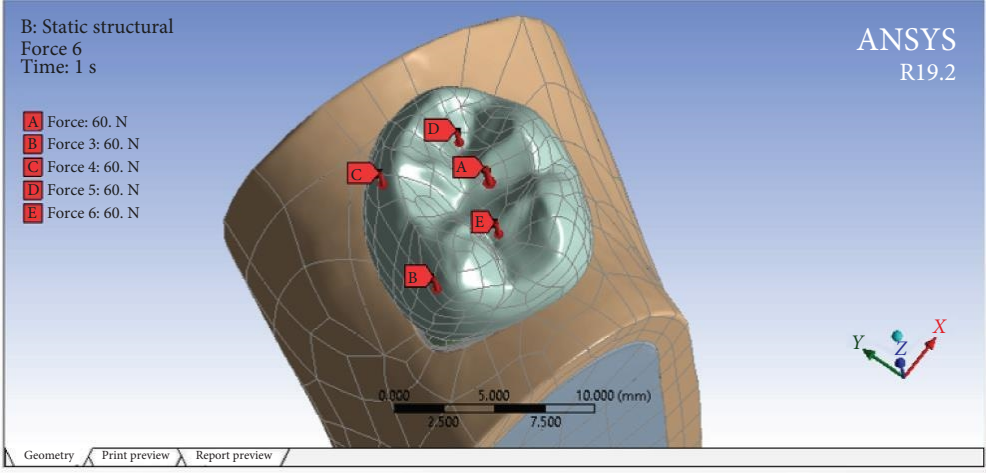
Applied loads.

The format of the designed models was converted from STL to standard for the exchange of product model data (STEP) using Geomagic software (Design X v2022). The models were subsequently uploaded to Ansys software (Workbench 19.2) for analysis. The interface between different layers in each model was treated as bonded. The meshing utilized 1,130,186 tetrahedral elements and 766,357 nodes for each model in all layers (Figure 3). The boundary conditions were defined by securing the lower portion of the mandible bone. To mimic occlusal load, a resultant vertical force of 300 N was distributed across five points (60 N at each point), with two points located on the external surface of the buccal cusps and three points on the internal surface of the lingual cusps [15, 16] (Figure 4). The Von Mises equivalent stress distribution was determined following the analysis.

## 3. Results

Table 2 illustrates the models' maximum Von Mises Stress (VMS) values in the enamel, dentin, bone, PDL, cementum, endocrown, and DME layers.

The MIT model, serving as the intact tooth reference, exhibited lower maximum stresses than the other models, with the highest stress values recorded in the MWD model, which lacked a DME layer. The maximum VMS occurred at the loading points, particularly around the CEJ, a common stress concentration area across all models.

**Figure 5:**
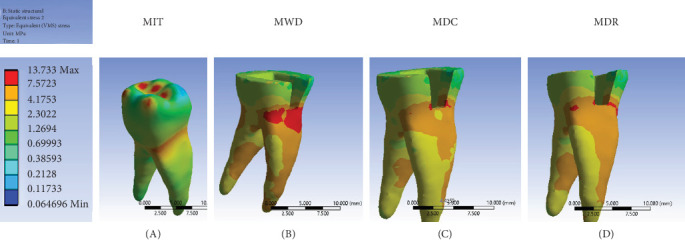
Stress distribution in dentin of models: (A) model of intact tooth; (B) model with endocrown and without DME; (C) model with endocrown + DME (composite flow); and (D) model with endocrown + DME (RMGI). DME, deep margin elevation; RMGI, resin-modified glass ionomer; VMS, Von Mises stress.

Analysis of the MIT indicated that stress was predominantly localized near the CEJ regardless of the occlusal load points. In the MWD, MDC, and MDR models, stress in the dentin layer increased relative to the MIT model. Notably, the MWD model demonstrated significant stress concentration beneath the mesial box within the dentin layer (Figure 5).

Considering that the ultimate compressive stress for dentin is 297 MPa [17], the peak stress observed in each model was substantially below this threshold, thus remaining within the dentin's tolerance limits.

**Figure 6:**
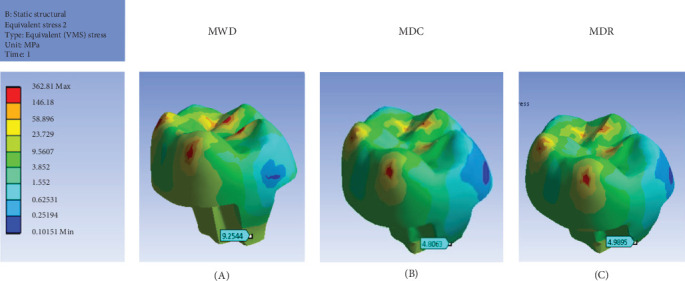
Stress distribution in endocrown restoration of models: (A) model without DME; (B) model with DME (composite flow); and (C) model with DME (RMGI). DME, deep margin elevation; RMGI, resin-modified glass ionomer; VMS, Von Mises stress.

The restored models (MWD, MDC, and MDR) followed a similar stress distribution pattern, with stress concentrations where the forces were applied. Among these, the MWD model experienced the highest levels of stress (Figure 6). Nevertheless, the maximal stresses were well within the handling capacity of all models, with no failures in the restorations when compared to the ultimate compressive strength of IPS e.max material, approximately 440 MPa [18].

The stress distribution patterns in the PDL and bone showed no significant variation due to the different model variables and were consistent across all scenarios.

**Figure 7:**
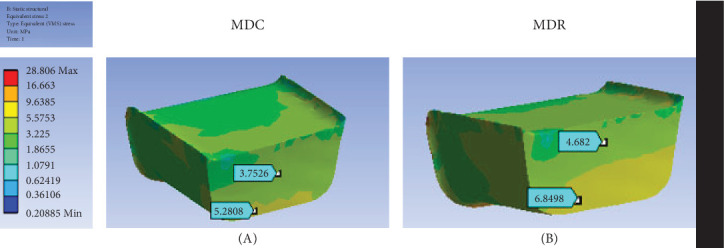
Stress distribution in the DME layer: (A) composite flow and (B) RMGI. DME, deep margin elevation; RMGI, resin-modified glass ionomer; VMS, Von Mises stress.

The flowable composite and RMGI used in the DME layer displayed similar stress distribution patterns, with higher stresses on the gingival than on the occlusal side (Figure 7). The stress values for these two materials were close, based on measurements at two randomly selected points on this layer. The slight differences in stress values can be attributed to the distinct elastic moduli of the materials.

The ultimate compressive strengths of RMGI and flowable composite are 214 MPa [19] and approximately 225 MPa [20], respectively. Given these properties, the maximum stress in both materials was significantly lower than their ultimate stresses, confirming the structural integrity under the applied conditions.

## 4. Discussion

The literature extensively examines DME used in restorations [4, 21–23]; however, few studies have investigated its impact on the biomechanical behavior of endocrowns and the surrounding tooth structure. This study aimed to evaluate the biomechanical responses of two restorative materials, flowable composite and RMGI, and to assess the influence of the DME technique on stress distribution in glass–ceramic endocrowns and the residual tooth structure.

The objective was to aid dentists in selecting appropriate materials and deciding whether to use the DME technique when encountering deep marginal caries below the CEJ. Since direct testing within a patient's mouth is impractical, FEA was employed to simulate the performance and identify the optimal material. Previous studies have already explored these aspects to some extent [4, 22, 23].

The findings from the FEM analysis indicated that using the DME technique reduces maximum stress levels. In this study, both flowable composite and RMGI were utilized for DME; they exhibited similar properties that contributed to decreased stress within the tooth layers and the restoration itself. Consequently, these results supported rejecting the first null hypothesis but affirmed the second.

The FEA was chosen for this study due to its advantages over traditional experimental testing, including repeatability without needing multiple samples, no ethical concerns, and cost-effectiveness. Additionally, FEA enhances understanding of the failure processes, unlike experimental studies, which only document the final failure outcome [24].

The results supported Rodrigues' [21] assertion that the cervical region of an intact tooth under occlusal force experiences the highest stress. As the enamel thins from the occlusal to the gingival part, it bears more significant stress, particularly around the CEJ area. The orientation change of enamel rods near the dentin enamel junction (DEJ), which are parallel in areas away from the CEJ and possess greater stress tolerance, also contributes to increased stress at this location [25].

According to the FEA outcomes, the MWD exhibited higher stress levels than those with a DME layer (MDC and MDR). Baldi et al.'s [4] research, which investigated stress around the CEJ and the pulp chamber floor, supports the findings that a DME layer significantly reduces stress at these sites. Grassi et al.'s [23] study concluded that using DME with ceramic restorations lowers stress, whereas using it with composite restorations does not alter stress levels, likely due to the similar elastic modulus between the crown and DME layer. However, the study utilized materials with a low elastic modulus for the DME layer.

The MIT model, applying occlusal force to an intact tooth, underwent less stress than the other models, suggesting that stress increases with the extent of tooth preparation and restoration.

This study used materials that closely match the tooth structure's elasticity in the DME layer, enhanced stress transmission to the underlying layers, and achieved a more uniform stress distribution in the dentin, thereby, reducing overall stress [22]. The DME layer acted as a stress absorber, preventing excessive stress transfer to the underlying structures. Chen, Lin, and Hou [22] and Grassi et al. [23] also observed similar stress absorption properties in flowable composite used as a DME material.

The stress distribution within the DME layer of both flowable composite and RMGI was within acceptable limits relative to their ultimate compressive strengths, corroborating Chen, Lin, and Hou's [22] findings that the maximum stress in the DME layer was well below its failure threshold.

Since our primary objective was to evaluate the primary materials—composite and RMGI—as their mechanical performance in the context of DME, we opted to omit the bonding layer below the composite layer. Existing literature indicates that the presence of a bonding layer, even at varying thicknesses, does not significantly affect the stress distribution on both the tooth and composite resin [12]. Therefore, to streamline the analysis and concentrate on the impact of these two materials, we opted to omit the bonding layer below the composite layer. This approach aligns with many prior studies that have similarly excluded the bonding layer from their models [4, 22].

In our study, we employed a tetrahedral mesh for the entire assembly to maintain a uniform modeling approach, consisting with other studies in the field [4, 22]. This choice was driven by several factors:

  - Uniformity and compatibility: Using a tetrahedral mesh across all layers simplifies the meshing process and ensures compatibility between different materials. Tetrahedral elements are commonly used for meshing complex geometries due to their flexibility in fitting irregular shapes.
  - Mesh density: We ensured that the mesh density was sufficient to capture critical stress distributions and mechanical behaviors throughout the model. While the same mesh type was used for all layers, we carefully selected the overall mesh size to balance computational efficiency with accuracy.
  - Validation: Our results indicate that this approach effectively captures the essential mechanical responses of the tooth structure under various loading conditions, demonstrating the adequacy of our meshing strategy. Also, Figure 3 that shows mesh refinement has been added to the document.

In all models, the stress levels in the PDL layer and the cortical and cancellous bones were consistent and showed no variation. Thus, the results of this study indicate that the DME layer primarily affects the tooth's cementum layer, with subsequent force transformation and stress distribution to other layers remaining unchanged.

Despite FEA's advantages in closely simulating various clinical scenarios, it has limitations, such as the assumption that the cement creates perfect adhesion between dental tissues and the restoration and that all materials are homogenous and isotropic is not replicable in vivo. This study assessed stress distribution under vertical static loads. Further clinical evaluations and in vitro research under different loading conditions are necessary.

## 5. Conclusion

Based on the results of this FEA study, the following conclusions were drawn:

1. Maximum stress location: An intact tooth's CEJ region consistently experiences the highest stress levels.
2. Stress reduction with DME: Employing DME using materials with an elastic modulus similar to dentin significantly reduces tooth structure stress.
3. Effectiveness of DME materials: When used as DME materials, there is no significant difference in stress reduction between the two tested materials, flowable composite, and RMGI. Both materials are effectively comparable in their performance.

These conclusions underscore the biomechanical advantages of using DME techniques in restorative dentistry, particularly with materials that closely mimic the mechanical properties of natural dentin. This approach not only enhances the longevity of the restoration but also preserves the tooth's structural integrity.

## Figures and Tables

**Table 1:** Mechanical properties of materials and tooth structures.

Material	Elastic modulus (GPa)	Poisson ratio
Enamel	84.10	0.33
Dentin	18.60	0.31
Pulp	0.0068	0.45
PDL	0.07	0.45
Cortical bone	13.7	0.30
Trabecular bone	1.37	0.30
Gutta percha	0.07	0.40
IPS e.max CAD; ivoclar vivadent/lithium disilicate glass ceramic	100.00	0.20
RMGI (GC fuji ll LC2; GC)	10.80	0.30
Universal flow resin composite (GC) [13]	7.90	0.28
Panavia F2; Kuraray Medical Inc [14]	12.00	0.33

Abbreviations: CAD, computer-aided design; IPS, innovative pressed system; LC, light cured; PDL, periodontal ligament; RMGI, resin-modified glass ionomer.

**Table 2:** Results of FEA and maximum VMS in different layers of models and tooth structure.

Maximum VMS (MPa)	MIT	MWD	MDC	MDR
DME	×	×	√	√
Material	—	—	Composite	RMGI
Entire assemble	271.83	362.81	351.14	351.14
Endocrown	—	362.81	352.81	351.14
Dentin	9.14	21.13	15.82	13.73
Enamel	120.1	191.04	163.35	163.31
Luting cement	—	34.9	19.99	19.99
Cementum	50.09	60.61	53.39	53.82
PDL	5.06	5.08	5.04	5.04
Cortical bone	21.05	21.55	21.05	21.05
Spongy bone	3.43	3.44	3.40	3.40

Abbreviations: DME, deep margin elevation; FEA, finite element analysis; MDC, model with DME of composite; MDR, model with DME of RMGI; MIT, model of intact tooth; MWD, model without DME; PDL, periodontal ligament; RMGI, resin-modified glass ionomer; VMS, Von Mises stress.

## Data Availability

The data that support the findings of this study are available from the corresponding author upon reasonable request.
